# The London Panel Committee

**Published:** 1914-11-28

**Authors:** 


### The London Panel Committee.

RELATIONS WITH PHARMACISTS.

At a meeting of the London Panel Committee on November 24 the chairman, Dr. H. J. Cardale, reported on the emergency action taken in the matter of the drug tariff. He said that after the new tariff had been agreed to by the chemists, the Commissioners suggested, and practically insisted, that there should be a selection of ten stock mixtures which should be placed on the tariff. The pharmacists asked the Panel Committee to take part in a conference with the Insurance Committee. He did not think that the Panel Committee could express a definite opinion. After a conference it was decided that it would be fair that the new tariff containing the clause with regard to stock mixtures should be accepted by the chemists, the Panel Committee agreeing on its part to delay the selection of mixtures during the lifetime of the present Committee—viz., until July next. The alternative would have been that the chemists would refuse to work under the new tariff and practitioners would have to work on the old one with its various inconsistencies, losing also the value of the agreement on the question of *aqua destillata*, which represented a saving to panel practitioners of £15,000 a year. Having suffered themselves at the hands of dictatorial authorities, panel practitioners ought to stretch a point and give their sympathy to the pharmacists on this occasion. The Committee approved the proposed line of action.

Arising from the recent conference called by the Local Government Board on the subject of district nursing in London a Central Council has been formed, and Dr. R. Carter and Dr. E. Hudson were appointed upon it as representatives of the Panel Committee.

It was decided to ask the Insurance Committee to call a conference upon the question of emergency dispensing by practitioners, the desire of the Panel Committee being to secure that the system of rendering and paying accounts in such cases be simplified.

The Question of Complaints.

It was suggested by the Pharmacy Sub-Committee that the attention of the Insurance Commissioners be drawn to the fact that no machinery exists for dealing with a complaint by a practitioner against a chemist, or *vice versa*. The Committee decided to bring the matter to the notice of the Commissioners, but the words "or *vice versa*" were deleted, the possibility of a complaint by a chemist against a doctor being apparently inconceivable.

